# Age-Related Differences in the Perception of Robotic Referential Gaze in Human-Robot Interaction

**DOI:** 10.1007/s12369-022-00926-6

**Published:** 2022-09-24

**Authors:** Lucas Morillo-Mendez, Martien G. S. Schrooten, Amy Loutfi, Oscar Martinez Mozos

**Affiliations:** 1 Centre for Applied Autonomous Sensor Systems, Örebro University, Fakultetsgatan 1, Örebro, 702 81 Sweden; 2 Department of Psychology, Örebro University, Fakultetsgatan 1, Örebro, 702 81 Sweden

**Keywords:** Human-robot interaction, Aging, Non-verbal cues, Social cues, Gaze following, Referential gaze

## Abstract

There is an increased interest in using social robots to assist older adults during their daily life activities. As social robots are designed to interact with older users, it becomes relevant to study these interactions under the lens of social cognition. Gaze following, the social ability to infer where other people are looking at, deteriorates with older age. Therefore, the referential gaze from robots might not be an effective social cue to indicate spatial locations to older users. In this study, we explored the performance of older adults, middle-aged adults, and younger controls in a task assisted by the referential gaze of a Pepper robot. We examined age-related differences in task performance, and in self-reported social perception of the robot. Our main findings show that referential gaze from a robot benefited task performance, although the magnitude of this facilitation was lower for older participants. Moreover, perceived anthropomorphism of the robot varied less as a result of its referential gaze in older adults. This research supports that social robots, even if limited in their gazing capabilities, can be effectively perceived as social entities. Additionally, this research suggests that robotic social cues, usually validated with young participants, might be less optimal signs for older adults.

**Supplementary Information:**

The online version contains supplementary material available at 10.1007/s12369-022-00926-6.

## Introduction

Social robots have the potential to assist different populations to perform everyday activities, including patients [1–3], students and instructors [4, 5], and older adults [6–8]. Indeed, the current number of people over 65 years old will double by 2050 [9], and thus, older adults will represent one of the most important potential beneficiaries of social and assistive robots.

In contrast to non-embodied agents, an important advantage of embodied robots is their ability to produce social cues through body gestures. Therefore, it is interesting to study the role of these social cues in human-robot interaction (HRI), and in particular with older users. However, and despite the prevalence of research exploring the positive effects of social cues from robots [10, 11], few of these studies compare the effect of these cues between older participants and younger controls [8]. For instance, research in social cognition has shown impairments in gaze following with older age [12]. Gaze following consists of the ability humans have to identify where the others are looking at, to engage in joint attention [13]. This ability is directly linked to referential gaze, a social cue of paramount importance that consists of a combination of head and eye movements to point to a location in space to another person [13–15]. Referential gaze has successfully been used in robots that lack the ability to move the eyes [11, 16]. Some of those robots, like Pepper^1^ [17], are widely used in research on socially assistive robots [18] for older adults. Thus, there is a need to study the influence of robotic referential gaze on older users.

**Fig. 1:**
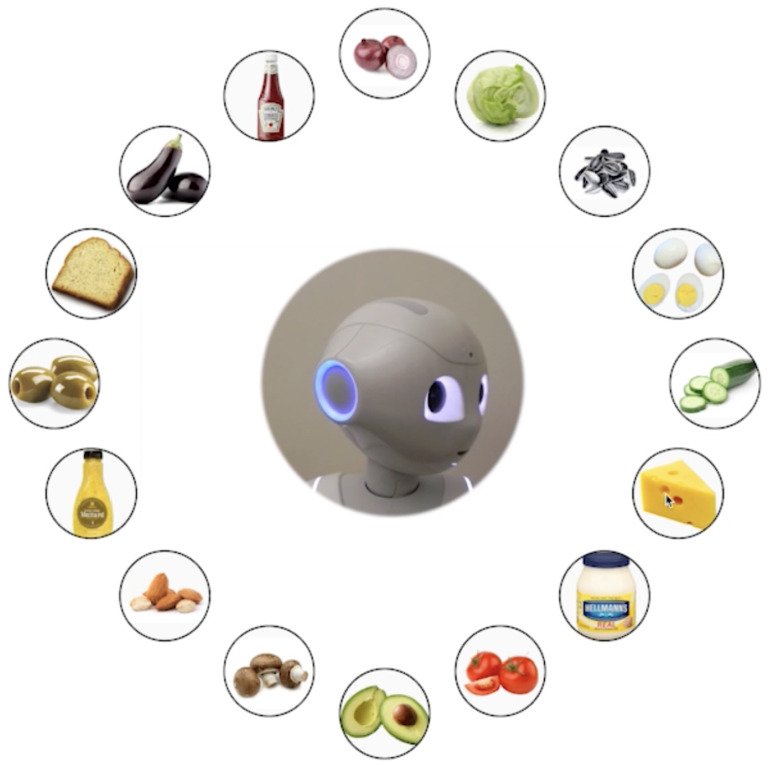
Snapshot of the online task consisting on preparing a sandwich. Verbal instructions were provided by a Pepper robot, either accompanied by referential gaze or static positioning towards the camera. This frame is part of a trial in which the robot mimics referential gaze towards the cheese, ingredient that the participant must click on

The benefits of studying referential gaze in socially assistive robots with older adults are twofold. First, it makes it possible to learn about the social responses that referential gaze from a social robot can evoke in this population, and thus, to gain more insight into human cognition [19, 20]. Given the expected decline in social cognition with older age, research on the impact of robotic referential gaze in older adults can reveal new insights about the perceived social nature of this cue in robots and see if this decline extends to non-human faces. Second, research on referential gaze from a widely available robot, like Pepper, serves to assess its communication capabilities in scenarios with older users. This research can inform the future design of social robots [21]. For instance, if the age-related decline of referential gaze extends to human-robot interactions, designers could create other cues that might prove more effective for older users. In summary, this research can have practical implications for the design of robotic assistance.

The work presented in this paper explores potential age-related differences in the perception of referential gaze from a social robot during a collaboration inspired by a daily life activity. To do so, we created an online task consisting of making a sandwich with the guidance of a robot. The task featured a video recording of a Pepper robot that remained static while looking towards the camera at all times, or moved its head towards an ingredient to initiate referential gaze (Fig. 1). We compared three age groups, namely *young adults*, *middle-aged adults*, and *older adults*, in task performance as well as self-reported social perception of the robot. We hypothesized that there would be differences in these measures among the groups of age. A final sample of 377 participants was included in the analyses. The remote nature of the study was an imposition stemming from the effects of the COVID-19 pandemic and the impossibility of performing face-to-face studies.

This paper extends our previous preliminary study [22] by the analysis of an extended data set of more than one hundred new participants. Crucially, the new data altered the data set qualitatively, as it permitted the creation of three age-groups similar in size and age distribution that led to new analyses. Finally, this new division provided essentially new insights in age-related differences in the perception of referential gaze.

## Related Work

There is extensive research on referential gaze in HRI (for two reviews on this, Ruhland et al. [23] and Admoni & Scasellatti [24]), as well as research on gaze following in older adults (for a review, see Zafrani et al. [8]). Nevertheless, to the best of our knowledge there is not published work involving both, with the notable exception of Pavic et al [25]. In this section, we will introduce these topics separately, as well as the use of online studies in HRI.

Robotic eye gaze is a well-established topic in HRI. Appropriate artificial gaze cues from robots have repeatedly shown a positive impact on the human partner. For instance, listeners’ recall of the content of a story told by a robot can be improved if accompanied by the corresponding gaze from the robot towards the listeners [26]. Similarly, robotic gaze has been shown to regulate participants’ roles during conversations [10]. Gaze aversion can also be effectively performed by a robot by moving its head away from the human, eliciting a perception of thoughtfulness on the latter [27].

In scenarios where a robot provides assistance, its referential gaze towards objects can help users to find those objects [24], although the benefits vary among studies. The work of Kontogiorgos et al. [28] explored the influence of eye gaze during a situated human-agent collaboration. They found that interaction times with an embodied agent were higher when this used non-verbal cues such as referential gaze, leading to a higher engagement with the agent in contrast to conditions not involving these cues. Nonetheless, Mwangi et al. [16] did not find that referential gaze from a robot moving its head affected the completion time of the task, although it did help to reduce the error rate. This result aligns with the research of Admoni et al. [11], who found robotic referential gaze to be a useful social cue during challenging tasks in contrast to simple ones.

The mixed results from previous research suggests context-dependent outputs from robotic referential gaze, similarly to human gaze [15, 29]. Although there seems to be a clear benefit in equipping robots with referential gaze, regardless of their degrees of freedom, the role of robot gaze as a social stimulus is not yet fully understood. Recent research has shown that making eye-contact with a robot engages different brain areas as compared to making eye-contact with humans, namely the right temporal parietal junction and the dorsolateral prefrontal cortex [30]. Nevertheless, making eye-contact with a robot can also evoke similar physiological responses as eye-contact between humans [31]. There is also behavioral evidence of social perception evoked by robots during a non-predictive cueing procedure. Participants showed faster reaction times towards the location gazed at by the robot if eye-contact was made immediately before [32]. Finally, although infants are sensitive to human features embedded in a robot, such as eyes and face, they do not react to referential gaze from robots, suggesting that the reflexive following of robotic gaze would be generalized from human gaze at a later stage in life [33].

As highlighted in the introduction, social robots will be at the service of an ageing population. However, research in human cognition has shown that social cognition deteriorates naturally as people age, independently of broader cognitive or perceptual decline [12, 34, 35]. There is evidence of age-related changes in the way social stimuli are perceived [12, 34, 36]. These range from a decline in emotion recognition [37–39] to difficulties in the identification of intentions and thoughts from others [13, 40] in normal ageing. Gaze following, required to initiate joint attention [13], deteriorates with age [12, 36]. To the best of our knowledge, there is just one published study addressing the relationship between age and the perception of the gaze of an embodied conversational agent [25]. Their results suggest that this decline is also present with virtual entities that resemble a human.

Exploring the age-related decline in gaze following with the use of robotic social cues would allow us to understand more about the true nature of social robots from the lens of social cognition. Additionally, it would inform about the effectivity of certain robotic cues for different user profiles. While the former approach is not new [19], it frequently used a more sophisticated robot, the iCub [41], capable of more complex behaviors given its degrees of freedom. Our research employs a Pepper robot [17], lacking eye movement, to comply with the last approach by learning more about a system available for the general public.

In this study, we designed an online task aimed at mimicking a real interaction between a human and a Pepper robot. This remote approach permitted to control the influence of some extraneous variables on the main outcome variables. One example is the effect of social presence caused by the robot looking at the user, which may lead the users to start a conversation with the robot and translated to higher completion times as in Kontogiorgos et al. [28]. Other HRI studies have also used online tasks [42, 43], and remote research measuring reaction times have demonstrated to be reliable when clear instructions are provided [44, 45]. An online, remote research approach is not surprising given the growing reach of the internet access worldwide, even among older adults. In particular, this approach offers the opportunity to participate from home to those older people with reduced mobility, difficulties to travel freely, or both, as those living in retirement homes. Finally, online research makes it easier to collect more data in less time, enabling a higher statistical power [46]. This is important, as small samples in HRI research have often led to underpowered studies [47].

## Aim of the Study

During the current task, a Pepper robot [17] guided participants of different age groups in preparing a sandwich by searching and clicking on the ingredients. There were two variations of the task corresponding to variations of the robot’s behavior: A *verbal only robot*, who gave verbal instructions and always looked to the camera during the task, and a *gaze robot*, who in addition to verbal instructions also moved its head towards the instructed ingredient. Referential gaze was elicited by making all head movements correspond to the verbalized ingredients, although participants were not informed about this.

We applied a 2x3 quasi-experimental mixed design with two robot conditions, *gaze* and *verbal only* (within-subject), and the age groups, *young adults* (YA), *middle-aged adults* (MA) and *older adults* (OA) (between-subjects). The age groups were formed based on Levinson’s stages of adult development [48] and the average working retirement age of 65. The age ranges were 18-44 years for *young adults*, 45-64 years for *middle-aged adults*, and 65-88 years for *older adults*.

**Table 1:** Final sample description (I), N=377

Group	Age (years)				Gender			Comfort w/ computers			Familiar w/ Pepper		
	Min	Max	Mean	SD	M	W	Other	No	Not Sure	Yes	No	Not Sure	Yes
OA	65	88	69.4	3.8	74	65	0	1	5	133	96	26	17
MA	45	64	58.2	4.8	36	66	1	3	9	91	65	20	18
YA	18	44	25.1	6.1	67	64	4	3	4	128	47	35	53

In our previous study [22], we discussed that the age distribution of the group under 65 years was too broad and could have occluded the appearance of an age-related effect of gaze following (18-64 vs. +65). In this study, we expected an age (YA, MA, OA) x robot condition (*gaze* vs *verbal only*) interaction based on previous research [12]. A different time facilitation, both in reaction times and task-completion times, between age groups caused by the use of robotic referential gaze would suggest the use of social cognition mechanisms to process this cue. Moreover, these mechanisms would be activated in the absence of eye movement. Finally, this result would suggest that simulating human cues in robots is not necessarily an efficient design strategy if aimed for older adults. We performed additional analyses to explore the perception of the robot in self-reported questionnaires.

## Methods

### Participants

A total of 422 participants completed the study. 10.7% of the participants were excluded from the analyses either due to incomplete data in age (n = 8); self-reported technical problems (n = 3); and unreliability of their mean delays between the onset of the trial and the appearance of the stimuli (n = 34, see Sect. 4.5). Participants were recruited from Spanish universities attending adult and regular education programs. We distributed the call through their designated mailing lists. Participation was voluntary and approval for distribution was obtained from the corresponding coordinators at each university. Inclusion criteria were having normal or corrected-to-normal vision, being fluent in Spanish, and being cognitively healthy.

**Table 2:** Final sample description (II)

Group	Completed education			
	High School	Bachelor’s	Master	PhD
OA	35	72	17	7
MA	26	61	8	6
YA	63	39	24	6

Tables 1 and 2 summarize the characteristics of the participants included in the analysis. We performed a G*Power analysis [49] with the final sample to calculate the statistical power of our experiment [*within-between interaction*; $N=377$; partial $\eta ~^2=0.06$]. The analysis returned a power of $1-b=0.99$, indicating a high probability of finding a significant interaction if it existed.

**Table:** 

Abbreviations for Tables 1 and 2:	
Young adults	[YA]
Middle-aged adults	[MA]
Older adults	[OA]
Standard Deviation	[SD]
Men	[M]
Women	[W]

### Stimuli

**Fig. 2:**
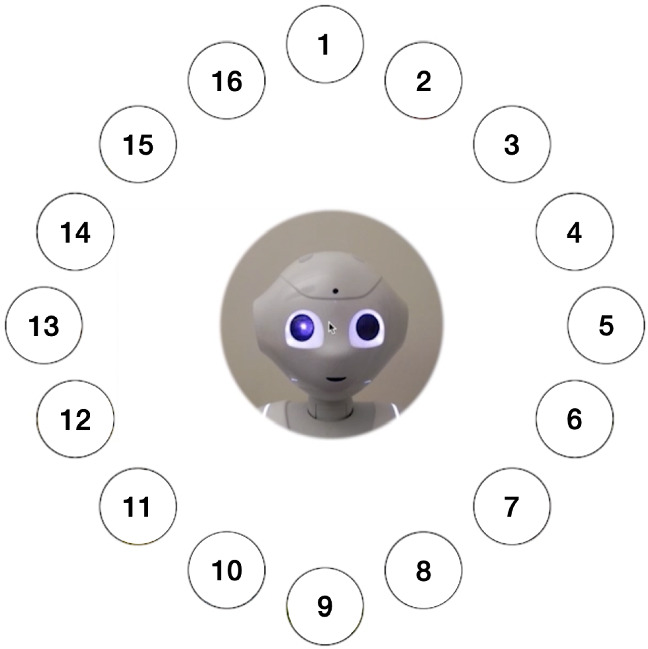
Numerical representation of each ingredient during the task

The task featured a central video of Pepper surrounded by sixteen ingredients. For the *gaze robot* condition, five head movements from Pepper were created as a baseline and were video recorded. These were designed using the pitch and yaw degrees of freedom of Pepper’s head^2^. In the task, position one corresponded to the top ingredient (just pitch movement). Position five was located on the robot’s left (clockwise, just yaw movement). Positions two to five were combinations of these two movements. Head movement towards these were tuned by varying pitch and yaw until they allowed discrimination between near ingredients. The rest of the positions (six to sixteen) and head movements are mirrored versions of the original ones in the imaginary horizontal and vertical axes that cross the center of the circle (See Fig. 2 for locations). The ingredient was mentioned last in every sentence to ensure that the end of the head movement towards the ingredient and the last syllable of the ingredient co-occured (See Supplementary Information to see the sentences and the corresponding yaw and pitch angles).

### Procedure

**Fig. 3:**
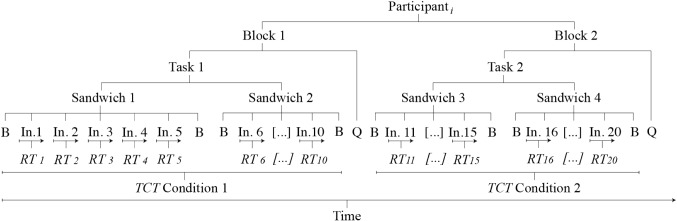
Structure of an experimental session: The letter ‘B’ stands for ‘Bread’, the number in ‘In. X’ represents the order of an ingredient, ‘Q’ refers to the questionnaire, ‘RT’ to ‘Reaction Time’ and ‘TCT’ to ‘Task-Completion Time’

For each variation of the task (i.e. for each condition), participants had to prepare two sandwiches following instructions from the robot, who named each ingredient one at a time before the user clicked on it. Within the task, the selection of an ingredient constituted a trial. For each condition, we measured task-completion and reaction times, among other questionnaires. The order of the sandwiches for each condition and order of the ingredients in each sandwich were fixed. The presented order of the blocks (task and questionnaire for each condition) was counter-balanced and participants were randomly assigned to one of the two possible orders (50.4% started with the *verbal only* condition). The main structure of the experiment is shown in Fig. 3.

Participants were first asked to wear headphones to minimize possible external noise and to calibrate the volume of the robot instructions until they heard them clearly. They also filled a brief test to ensure they could see the screen accurately and were requested to exit the study if they failed for this reason. All participants were encouraged to avoid distractions, to be rested before starting, to use a mouse device, and to refrain from talking about the study with other potential users. They were informed about the content, aim and procedure of the study, and the possibility of ending participation at any time. They gave written consent to participate in accordance with the declaration of Helsinki. After filling a demographic survey, they performed three trials in which instructions appeared in the middle as text, instead of the robot. All the ingredients and their names were shown to them to exclude the possibility of a participant not recognizing certain ingredients. Finally, participants were told that the study would last about 15 minutes and were encouraged to perform the tasks as well as they could. No personal data that allowed for their identification were obtained during the study.

### Materials

The task program, including the self-report questionnaires, was built using *Labvanced* [50], an online platform to design and distribute online experiments. All responses were registered in Labvanced.

Prior to the beginning of the task, participants filled a demographic survey in which they reported their nationality, age, gender (*man*, *woman*, *other*), completed education (*school*, *high school*, *bachelor’s*, *master*, *PhD*, *other*), comfort with computers, and familiarity with Pepper. After completing a task, participants completed the following set of self-report measures:

- The mental demand subscale of the NASA-Task Load Index (NASA-TLX) [51], a computerized 21-point slider [1=Low; 21=High] for assessing the mental demand of the task.
- A modified version of the anthropomorphism semantic differential subscale [5-points] from the Godspeed Questionnaire Series [52], to measure the perceived anthropomorphism of the robot. The scale was modified due to the irrelevance of an item within this study, (*moving rigidly-elegantly*). Moreover, we added the item *mechanical-organic*, as in [53]. The subscale was composed of five items in total.
- The Robotic Social Attributes Scale (RoSAS) [53], a 5-point likert scale [1=Totally disagree; 5=Totally agree], to measure the perception of warmth, competence, and discomfort caused by a robot. Each dimension was composed of six items.
- Q1: *Did you notice any difference between the robots in the tasks?* to check whether the person was aware of the difference between robot conditions. Participants could choose between *yes* and *no*, and were given some space to write what they thought the differences were. This question was made at the end of the study.
- Q2: *Which robot did you prefer from the ones you interacted with?* to check their preferred robot condition (*gaze* or *verbal only*). They were presented with two simultaneous videos of each condition and were asked to choose between *robot a* (corresponding to *verbal only*), *robot b* (corresponding to *gaze*), and *no preference*. This question was made at the end of the study.

### Behavioral Measures

We defined task-completion time as the sum of correct trial times per condition. A trial was correct when the ingredient mentioned by the robot was clicked. Reaction time was defined as the time span between the onset of the video frame when the robot starts naming an ingredient and the moment a participant clicked on that ingredient. Because of the remote nature of the study, there was a certain delay (in milliseconds) between the onset of the trial and the moment the stimuli appeared in the screen of a participant. This delay could somewhat differ between participants, for instance based on the computer capabilities or its operating system. For this reason, we corrected all reaction and trial times by subtracting the mean delay that each participant experienced during the study. Participants with mean delays that were not reliable, as indicated by their extreme standard deviations, were excluded from the analysis.

Because the selection of the ingredient *bread* was predictable, always first/last ingredient, we removed the corresponding trials from the analysis. Additionally, participants had to repeat incorrect trials for the same ingredient until it was correct or until they reached three incorrect attempts. In incorrect and consecutive trials with the same ingredient, reaction time was excluded from the analysis. The maximum number of correct trials per task was ten, corresponding to five ingredients for each of the two sandwiches.

## Results

The data was analyzed with the R software [54]. Due to violations of assumptions for the mixed ANOVA test, we analyzed the times and questionnaires using Mixed Robust ANOVA tests with the *WRS2* package [55, 56], using 20% trimmed means and 2000 bootstrapped samples. We used ${\hat{\xi }}$ as a measurement of the effect size to report the importance of significant results for the robust analyses [57, 58]. This measure is equivalent to Cohen’s *d* for t-tests or Partial Eta-squared ($\eta _{p}^{2}$) in ANOVA. Values of 0.10, 0.30, and 0.50 were taken to correspond to small, medium and large effect sizes respectively [56, 57]. All Post Hoc analyses had Bonferroni corrected adjusted p-values .

### Reaction Times and Task-Completion Times

We present the reaction times (RT) and task-completion times (TCT) for the two robot conditions: *gaze* (GR) or *verbal only* (VR); per age groups: *young adults* (YA), *middle-aged adults* (MA), and *older adults* (OA). To analyze the RT data, we used the median RT of the correct trials within each task per participant (Fig. 3). Incorrect trials, 2% of all, were excluded from the analysis.

**Fig. 4:**
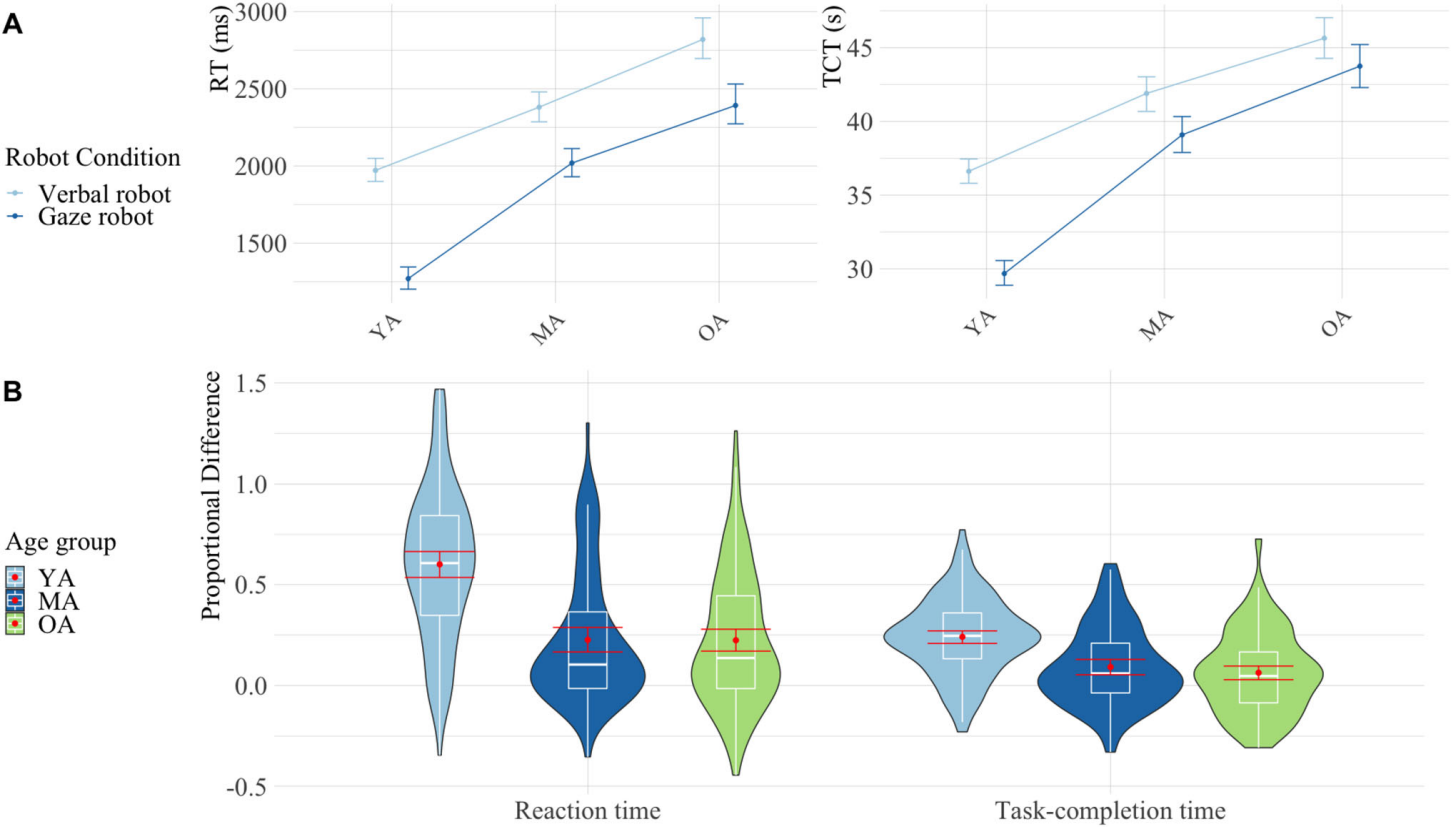
A) Mean Reaction Time (left) and Task-Completion Time (right) for each age group: younger adults (YA), middle-aged adults (MA), and older adults (OA). B) Violin plots with means in red of the proportional differences between robots. Six extreme values above 1.5 were removed to constrain the proportions of the RT graph, five from YA (1.54, 1.79, 2.34, 2.53 and 2.58) and one from OA (1.58). Error bars show 95% bootstrapped confidence intervals in all graphs

**Table 3:** Main/interaction effects on the times and *mean time* ± *standard deviation* for every level of the variables

	RT (ms)	TCT (s)	% Faci. (RT)	% Faci. (TCT)
**Age group**	$p<.001$ (***)	$p<.001$ (***)	$p<.001$ (***)	$p<.001$ (***)
YA	$1621 \pm 560$	$33.1 \pm 5.94$	$65 \pm 48$	$25.6 \pm 20.3$
MA	$2201 \pm 531$	$40.5 \pm 6.4$	$22.6 \pm 32.6$	$9.1 \pm 19.4$
OA	$2607 \pm 827$	$44.7 \pm 8.7$	$23.4 \pm 34.5$	$6.8 \pm 21.1$
**Robot**	$p<.001$ (***)	$p<.001$ (***)	–	–
VR	$2397 \pm 720$	$41.4 \pm 7.71$		
GR	$1890 \pm 768$	$37.4 \pm 9.26$		
Age*Robot	$p=.0105$ (*)	$p<.001$ (***)	–	–
YA$_{VR-GR}$	$702 \pm 469$	$6.94 \pm 5.5$		
MA$_{VR-GR}$	$363 \pm 526$	$2.82 \pm 7.21$		
OA$_{VR-GR}$	$428 \pm 742$	$1.9 \pm 8.65$		

The mean RT for the different age groups and robot conditions are shown in Fig. 4A (left). Means, standard deviations and p-values for RT are also reported in Table 3. There was a significant difference between the robot conditions, showing faster reaction times for the GR condition as compared to the VR condition (${\hat{\xi }}=0.47$). We also found a main effect of age (${\hat{\xi }}=0.81$). A Post Hoc analysis showed significant differences in RT between each age group pair, indicating slower RT with older age (all $p<.001$). We found an interaction effect between robot condition and age group, suggesting that the difference between the robot conditions was especially large in the YA (mean difference between VR and GR is 702 ms). We explored this interaction by performing a paired robust t-test to compare the robot conditions for each age group. All groups showed significantly faster reaction times for the GR condition compared to the VR condition (all $p<.001$), although the effect size was particularly large for YA (${\hat{\xi }}=0.9$; ${\hat{\xi }}=0.45$ for MA and ${\hat{\xi }}=0.4$ for OA). In order to take into account age-related differences in overall RT, we calculated the strength of the facilitation as a proportional difference score $(RT_{VR} - RT_{GR}) / RT_{GR}$, following previous research in spatial attention [12, 59]. A one-way Robust ANOVA showed a significant difference between age groups in the strength of the facilitation effect (${\hat{\xi }}=0.58$). A Post Hoc analysis indicated a significant difference in facilitation effect for RT between YA & MA and YA & OA (both $p<.001$), but not between MA & OA . In summary, reaction times were faster with the *gaze robot* than with the *verbal only robot*, especially in *young adults* (Fig. 4B and Table 3).

The mean TCT for the different age groups and conditions are shown in Fig. 4A (right). Means, standard deviations and p-values for TCT are also reported in Table 3. Completion times were significantly faster for the GR condition compared to the VR condition (${\hat{\xi }}=0.33$). We also found a main effect of age (${\hat{\xi }}=0.79$). A Post Hoc analysis showed significant differences in TCT between each age group pair, showing slower completion times with older age (all $p<.001$). We also found an interaction effect between robot condition and age group, suggesting that the difference between the robot conditions was especially large in the YA. We explored this interaction by performing a paired robust t-test to compare the robot conditions for each age group. The YA and MA groups showed significantly faster task-completion times for the GR condition compared to the VR condition (both $p<.001$), although this was not the case for older adults ($p=0.26$). The effect size was particularly large for YA (${\hat{\xi }}=0.82$; ${\hat{\xi }}=0.24$ for MA). The strength of the facilitation effect was also calculated as a proportional difference score $(TCT_{VR}-TCT_{GR})/TCT_{GR}$. A one-way Robust ANOVA showed significant difference between age groups in the strength of the facilitation effect (${\hat{\xi }}=0.48$). A Post Hoc analysis indicated a significant difference in facilitation effect for TCT between YA & MA and YA & OA (both $p<.001$), but not between MA & OA ($p<.054$). In summary, task-completion times became faster with the *gaze robot* compared to the *verbal only robot*, especially in *young adults*, although this difference was not statistically significant for the older group (Fig. 4B and Table 3).

**Table 4:** Cronbachs’s $\alpha$ for every social perception score; main/interaction effects on the social perception scores, and *mean score* ± *standard deviation* for every level of the variables

	Anth. ($\alpha$ = .88)	Warmth ($\alpha$ = .87)	Compt. ($\alpha$ = .85)	Discom. ($\alpha$ = .8)
**Age group**	$p<.001$ (***)	$p<.001$ (***)	$p=.014$ (*)	$p=.6$ (NS)
YA	$2.21\pm 0.85$	$2.16\pm 0.82$	$3.43\pm 0.84$	$1.72\pm 0.7$
MA	$2.76\pm 0.94$	$2.47\pm 0.79$	$3.61\pm 0.69$	$1.7\pm 0.6$
OA	$2.84\pm 0.88$	$2.5\pm 0.81$	$3.63\pm 0.735$	$1.74\pm 0.6$
**Robot**	$p<.001$ (***)	$p<.001$ (***)	$p<.001$ (***)	$p=.1$ (NS)
VR	$2.46\pm 0.94$	$2.25\pm 0.81$	$3.63\pm 0.73$	$1.76\pm 0.64$
GR	$2.72\pm 0.92$	$2.5\pm 0.82$	$3.73\pm 0.67$	$1.68\pm 0.62$
Age*Robot	$p=.05$ (*)	$p=1$ (NS)	$p=.4$ (NS)	$p=.7$ (NS)
YA$_{VR-GR}$	$-0.37\pm 0.7$	$-0.33\pm 0.65$	$-0.4\pm 0.67$	$0.1\pm 0.52$
MA$_{VR-GR}$	$-0.32\pm 0.78$	$-0.25\pm 0.68$	$-0.37\pm 0.75$	$0.15\pm 0.58$
OA$_{VR-GR}$	$-0.09\pm 0.68$	$-0.18\pm 0.63$	$-0.28\pm 0.62$	$0\pm 0.5$

**Table:** 

Abbreviations for Tables 3 and 4:	
Young adults	[YA]
Middle-aged adults	[MA]
Older adults	[OA]
Verbal only robot	[VR]
Gaze robot	[GR]
Reaction Time	[RT]
Task-Completion Time	[TCT]
RT facilitation effect (See 5.1)	[% Faci. (RT)]
TCT facilitation effect (See 5.1)	[% Faci. (TCT)]
Anthropomorphism	[Anth.]
Competence	[Compt.]
Discomfort	[Discom.]
$p<.05$	*
$p<.01$	**
$p<.001$	***

### Self-Report Measures

We present the scores of the self-report questionnaires and scales from Sect. 4.4. Table 4 shows the social perception mean scores, the Cronbachs’s $\alpha$ of each construct, and the corresponding main and interaction effects.

We found a significant effect of age for anthropomorphism (${\hat{\xi }}=0.4$), warmth (${\hat{\xi }}=0.27$), and competence (${\hat{\xi }}=0.19$). A Post Hoc analysis showed that OA perceived the robots as more anthropomorphic, competent, and warmer against MA and YA (both $p<.001$ vs. MA and YA in anthropomorphism and warmth; $p=.007$ vs. MA and $p=.043$ vs. YA in competence). We also found a significant effect of robot condition for anthropomorphism (${\hat{\xi }}=0.21$), warmth (${\hat{\xi }}=0.2$), and competence (${\hat{\xi }}=0.3$). All these scores were higher for the *gaze robot* condition. Finally, we found a significant interaction in anthropomorphism, showing a narrower score variation between robot conditions for OA as compared to MA and YA. This indicates that the older participants barely varied their anthropomorphism scores between the robot conditions (See Table 4).

Finally, the mean of the mental demand subscale from the NASA-TLX indicated a very low score for most participants ($M=3.7\pm 3.5$ out of 21 points). Along with the low trial error rate (2%) during the tasks, this was an indication of the low difficulty they involved. We did not analyze this data given its limited variability and the evident floor effect it showed [60].

### Additional Analyses

We repeated our main analyses for the subset of all participants who retrospectively reported to not have noticed the difference between the robot conditions by answering ’No’ to Q1 (see Sect. 4.4), and therefore, who might have been unaware of the head movement in the gaze robot (a total of 124, 32.8% of the sample). In this subsample, we found: (1) a main effect of age group on RT and TCT, both at $p<.001$, and a main effect of age group on anthropomorphism at $p=.012$. These results are similar to those of the total sample (N=377); (2) a main effect of robot condition on RT and TCT, at $p=.03$ and $p=.04$ respectively, also in the direction of the results analysis performed with the total sample; (3) a main effect of robot condition on discomfort at $p<.001$, scoring the GR 0.08 points higher in this dimension; (4) an over-representation of OA (51.1%), as compared to MA (36%) and YA (12%) ($\chi ^2(2, 372)=48.3, p<.001$).

Two hundred and fifty participants (66.3%) expressed a preference for a robot in their answer to Q2. 205 of them ($82\%$) preferred the GR over the VR. The difference in preference between age groups (GR in YA=85%, in MA=85.2%, and in OA=72%) was marginally significant ($\chi ^2(1, 250)=5.9, p = .052$), indicating a lower preference of the GR in older adults as compared to the other groups.

## Discussion

This work explored age-related differences in the perception of referential gaze from a social robot. We measured participants’ task-performance and social perception of a robot during a collaborative task of preparing a sandwich with a Pepper robot. Our main objective was to explore the different responses between three different age groups (young, middle-aged and older adults) towards the referential gaze from a robot. We examined task performance by measuring the time a participant took to click on an ingredient indicated by the robot (reaction time) and the time needed to finish the task (task-completion time). The data revealed that participants were faster when the robot used referential gaze than when the robot gave only verbal instructions. This was true both for reaction time (average of 500 ms or 21% faster) and task-completion time (average of 4 s or 9.7% faster). This effect occurred in every age group, but especially in the younger group. On average, young adults were 65% faster in their RTs when they were guided by the gazing robot. In contrast, middle-aged and older adults showed a smaller benefit of 22.6% and 23.4% respectively. These results were similar for the task-completion time, with younger participants completing the task 25.6% faster when guided by the gazing robot. Middle-aged and older adults showed a smaller benefit of 9.1% and 6.8% respectively, although it did not reach significance for the older group.

We measured age-related differences in the effect of robotic gaze on reaction time and task-completion time to explore possible effects in social cognition, but also to explore practical outcomes in the performance of a full task. Young adults showed a clear benefit due to robotic gaze in both these outcome measures, as compared to the older groups. First, these results suggest that the robotic referential gaze of a Pepper robot is effectively perceived as a social cue. We observed a similar age-related decline in reaction times as would occur if human referential gaze was used as cue during task performance [12, 36]. Second, this decline also manifests in real interactions and has an overall effect on the time it takes to end a task with a robot, i.e., the efficiency of the collaboration.

In our previous preliminary study [22], with the same procedure but limited sample (N = 276), we could not find an interaction between the robot condition and the age group. There are two plausible reasons for this. First, in [22] the participants were divided in two groups of similar size, but that were not very different in their mean age (53.4 and 69.3 years). Second, the younger group showed a much wider variability of ages than the older one (18-64 vs. 65-88). In the present paper, the new data permitted a finer division of age ranges and made a younger (18-44) and a middle-age (45-64) group emerge. With this division, the differences between the younger and the older groups became evident.

Our results also showed that on average, referential gaze had a positive effect on task-completion time. These results contrast with those of Kontogiorgos at al. [28] and Mwangi et al. [16]. Additionally, this effect on task-completion time occurred in a non-challenging task, in contrast to the work of Admoni et al. [11], which discussed that referential gaze is only useful in challenging task. The simplicity of this task was shown by the low error rate and mental demand reported by participants. One possible explanation for this discrepancy is related to the absence of a robotic social presence within the same shared spatial environment in our study, i.e. the use of a recorded video [61], and the limited interactive scenario we explored. While this reduced the external validity of this experiment, we could control and isolate our variables of interest in a trade-off between external and internal validity.

During the study, participants also reported their social perception of the robot. The measured domains were *anthropomorphism*, *warmth*, *competence*, and *discomfort*. We found an increase in all the social perception scores, except for discomfort, as a result of the referential gaze. Additionally, when asked about their preference, the robot using referential gaze was chosen as the favourite. This is in line with previous notions supporting that social behaviors improve the acceptance of robots [31, 62]. We also found that older adults perceived the robot as more anthropomorphic, competent, and warmer compared to the other groups. These results can be considered under the light of a novelty effect, as shown by the fact that the older group was less familiar with Pepper. The attribution of anthropomorphism to a robot is linked with novelty effects. It can help reducing the uncertainty associated with an unknown agent and making sense of their actions [63]. It is not surprising that warmth and competence, both based on a certain degree of anthropomorphism, were also higher for this group. Additionally, the lack of variation in discomfort between the age groups can also be explained by novelty effects. Carpinella et al. [53] showed that this dimension does not vary as an effect of familiarity. Finally, we found that the anthropomorphism of the robot varied less for older adults between the robot conditions. This was also the case for warmth and competence, although the variation did not reach significance between the groups. This small difference for older adults points in the direction of a different social perception of a robot, i.e. a limitation in the attribution of human characteristics towards it as a result of its social behavior.

Moreover, a proportion of $32.8\%$ of the participants reported not detecting the differences between the robot conditions. For these participants, there was also a time facilitation of referential gaze, suggesting a reflexive nature of robotic gaze following. Nevertheless, the magnitude of the facilitation remained equal between groups, both for reaction and task-completion times. This is not surprising given that most of the participants in this ’unaware’ group were older adults (57% older, 30% middle-aged, and 13% young adults). This unbalance between groups limits the statistical power to find main effects of age group and thus, interaction effects. However, the over representation of older participants is in line with the idea of an age-related decline robotic gaze following, even if purely reliant on head movement, as the majority of them reported not noticing differences between conditions. Nonetheless, the question about differences between conditions appeared at the end of the study. It cannot be excluded that the over representation of older participants reflects a broader cognitive decline or difficulties in remembering these differences [64].

This study used a Pepper social robot as main stimulus to initiate referential gaze. Previous research has showed an age-related decline in the perception of gaze from a virtual agent [25]. In this research, we extended this to a commercially available robot which is also lacking eye movement, contrary to a virtual agent. The choice of Pepper was determined by these two factors. Future research should include a clearly non-social signal to further investigate the social nature of referential gaze from a robot moving its head. The perception of general and biological motion also declines in normal ageing [65, 66]. Given the relatedness of motion perception and social cognition [67], the non-social signal to be used should also control for movement. This would permit to isolate the social component of referential gaze in Pepper as final cause of these results. Future studies should also explore the reflexive nature of robotic gaze following by manipulating the validity of the cues, set to 100% in the present one. The manipulation of gaze predictability can also help to study the effects of strategic gaze following, led by the characteristics of the task, and reflexive gaze following, modulated by the social nature of the stimulus [12]. Additionally, the potential appearance of attentional costs driven by the robot misleading the human could be studied, as they are not present for older adults when following human gaze [12, 36]. Although the addition of invalid cues would be difficult to justify within a purely validation scenario, more concerned with the direct utility of the robot, these can also be useful to explore the impact of credibility of the robot and trust in a system that might not always be completely accurate [68].

Besides gaze following, there are other plausible strategies to study social cognition in older adults during HRI. In addition to gaze following, there is a decline in emotion recognition in faces [35, 37–39] with older age. Emotion recognition can be investigated with robots with enough degrees of freedom to simulate facial cues suggestive of emotions. As in this research, this would be interesting, both to learn about the generalization of human responses towards social stimuli in different contexts and to understand the usefulness of robotic facial expressions for older adults. Moreover, future studies should attempt to replicate the current study by employing robots with average baseline social perception scores different to those of Pepper. Additionally, including robots in face-to-face scenarios is important to see how well these results generalize with the additional component of social presence.

There are two limitations in the generalizability of our results arising from our sample. First, the age groups also differed in their level of education and familiarity with Pepper (Tables 1 and 2). Although expected, these differences prevent strong conclusions in terms of only chronological age. This is important to acknowledge, as variables such as familiarity might have a role in the perception of gaze from a social robot. It could occur that those more familiarized with social robots perceive them as more anthropomorphic and as social entities, potentially showing higher facilitation effects irrespective of age. To understand the role of chronological age in the perception of social robots, longitudinal research can help isolating other confounding variables. Alternatively, quasi-experimental research should aim to reach a high homogeneity between age groups in these potentially confounding variables. For example, an introductory video of the robot showing its capabilities could be shown to all participants prior to the experiment to familiarize with it. Second, there was an under representation of men in the *middle-aged adults* group in our sample. However, most differences in this research occurred between *young adults* and older ones, with a balanced representation of gender.

## Conclusion

We explored the influence of referential gaze from a Pepper robot in three age groups: *young adults*, *middle-aged adults*, and *older adults*. A facilitation effect of referential gaze was found in all the groups, especially in *young adults*. *Older adults* still reacted to the instructions 23.4% faster when the robot used referential gaze than when the robot only gave verbal instructions. Although they also completed the task 6.8% faster, this gain was not statistically significant. Overall, head movement representing referential gaze seems to be beneficial for task performance. Additionally, the attenuation of this benefit in task performance with older age suggest that Pepper is probably perceived as a social entity, and that social cues might be less optimal for older users. Moreover, average social perception scores increased in response to referential gaze. However, *older adults* showed a smaller difference in perceived anthropomorphism between the robot conditions than to *middle-aged adults* and *young adults*, indicating a lower attribution of social traits to the robot in response to its behavior. Finally, we found differences between the age groups in their perception of how anthropomorphic, warm and competent they perceived the robot (regardless of the robot condition). These group differences are more likely explained as a result of novelty effects than chronological age.

Future research should better isolate the role of chronological age in the perception of social robots. Additionally, it should include non-social control cues to better inform the possible differences between human and robot gaze cues. Finally, adding invalid cues would be convenient to explore potential attentional costs and to determine the role of trust in the interaction. This user-centered approach would be of value to inform future designs of non-verbal cues in HRI that lead to a wider acceptance of social robots.

## Supplementary Information

Below is the link to the electronic supplementary material.

Supplementary file 1 (pdf 379 KB)

Supplementary file 2 (mp4 20240 KB)

## Data Availability

The data generated for this experiment are openly available at: 10.17605/OSF.IO/V3GP5

## Footnotes

1 https://www.softbankrobotics.com.

2 http://doc.aldebaran.com/2-4/family/robots/joints_robot.html.
