# Supplementary material for: Age-Related Differences in the Perception of Robotic Referential Gaze in Human-Robot Interaction
Source: Int J Soc Robot. 2022 Sep 24:1–13. Online ahead of print. doi: 10.1007/s12369-022-00926-6 (PMC9510350; doi:10.1007/s12369-022-00926-6)
Supplement: Supplementary file 1 — (pdf 379 KB) [file 12369_2022_926_MOESM1_ESM.pdf]

Age-Related Differences in the Perception of Robotic Referential Gaze in HRI

Supplementary Information

Lucas Morillo-Mendez[1*], Martien G.S. Schrooten[2], Amy Loutfi[1] and Oscar Martinez Mozos[1]

[1*]Centre for Applied Autonomous Sensor Systems, Örebro University, Fakultetsgatan 1, Örebro, 702 81, Sweden.
[2]Department of Psychology, Örebro University, Fakultetsgatan 1, Örebro, 702 81, Sweden.

*Corresponding author(s). E-mail(s): lucas.morillo@oru.se;

This supplementary material is extended with an example video (*task_video*) and the collected data, which can be found at: DOI10.17605/OSF.IO/V3GP5

Pitch and Yaw Angles for Pepper's Head

Five head movements were created towards the position of each ingredient for the Gaze Robot condition (GR). These were designed using the pitch and yaw degrees of freedom of Pepper's head[1]. In the task, position one corresponded to the top ingredient (only pitch movement; see fig.1). Position five was located on the robot's left (only yaw movement). Positions two to four were combinations of these two movements. Head movement towards these were tuned by varying pitch and yaw until they allowed discrimination between near ingredients. The rest of the positions (six to sixteen) and the corresponding head movements are mirrored versions of the original ones in the imaginary horizontal and vertical axes that cross the center of the circle (see table 1).

[1]http://doc.aldebaran.com/2-4/family/robots/joints_robot.html

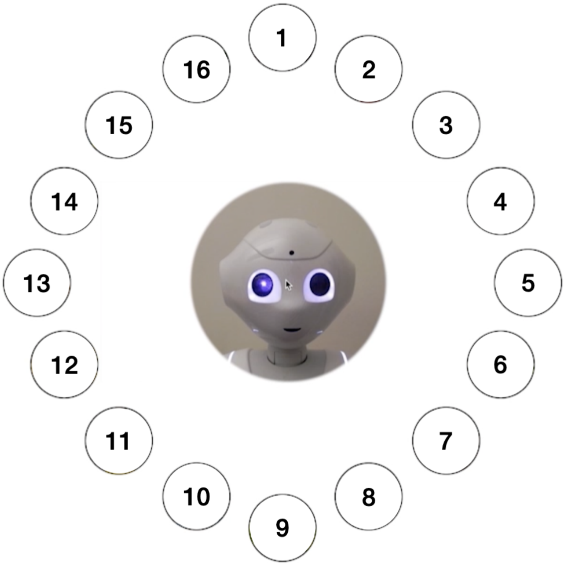

Fig. 1 Position of the ingredients during the task.

Table 1 Pepper set of head movement towards positions

Position Nr	Yaw (°)	Pitch (°)	Yaw (Rad)	Pitch (Rad)
1	0	-30	0.000	-0.524
2	17.5	-22.5	0.305	-0.393
3	35	-15	0.611	-0.262
4	52.5	-7.5	0.916	-0.131
5	70	0	1.222	0.000
6	52.5	7.5	0.916	0.131
7	35	15	0.611	0.262
8	17.5	22.5	0.305	0.393
9	0	30	0.000	0.524
10	-17.5	22.5	-0.305	0.393
11	-35	15	-0.611	0.262
12	-52.5	7.5	-0.916	0.131
13	-70	0	-1.222	0.000
14	-52.5	-7.5	-0.916	-0.131
15	-35	-15	-0.611	-0.262
16	-17.5	-22.5	-0.305	-0.393

Ingredient Identity and Instructions Transcription

Some of the available ingredients varied among recipes. For a list of all ingredients and transcription of the instructions of the robot see tables 2-5.

Table 2 Verbal only robot (VR) - Recipe 1

Ingredient	English transcription	Ingredient position
Bread	"First, click on the bread"	7
Butter	"Now, click on the butter"	10
Pepper	"For the next step, click on the pepper"	8
Tomato	"Now click on the tomato"	14
Walnuts	"Click on the walnuts"	13
Spinach	"Next, click on the spinach"	6
Bread	"To conclude, click on the bread"	7

Table 3 Verbal only robot (VR) - Recipe 2

Ingredient	English transcription	Ingredient position
Bread	"First, click on the bread"	16
Mayonnaise	"Now, click on the mayonnaise"	12
Lettuce	"For the next step, click on the lettuce"	3
Egg	"Now click on the egg"	7
Sunflower Seeds	"Click on the sunflower seeds"	6
Mushrooms	"Next, click on the mushrooms"	10
Bread	"To conclude, click on the bread"	16

Table 4 Gaze robot (GR) - Recipe 1

Ingredient	English transcription	Ingredient position
Bread	"First, click on the bread"	3
Ketchup	"Now, click on the ketchup"	13
Onion	"For the next step, click on the onion"	7
Cucumber	"Now click on the cucumber"	12
Egg	"Click on the egg"	6
Mustard	"Next, click on the mustard"	5
Bread	"To conclude, click on the bread"	3

Table 5 Gaze robot (GR) - Recipe 2

Ingredient	English transcription	Ingredient position
Bread	"First, click on the bread"	14
Cheese	"Now, click on the cheese"	6
Eggplant	"For the next step, click on the eggplant"	15
Olives	"Now click on the olives"	13
Tomato	"Click on the tomato"	8
Almonds	"Next, click on the almonds"	10
Bread	"To conclude, click on the bread"	14
